# Multifunctional substrate of label-free electrochemical immunosensor for ultrasensitive detection of cytokeratins antigen 21-1

**DOI:** 10.1038/s41598-017-01250-0

**Published:** 2017-04-21

**Authors:** Huiqiang Wang, Xin Gao, Zhanfang Ma

**Affiliations:** Department of Chemistry, Capital Normal University, Beijing, 100048 China

## Abstract

Poly(thionine)-Au, a novel multifunctional substrate with excellent redox signal, enzyme-like activity, and easy antibody immobilisation, was synthesised using HAuCl_4_ as the oxidising agent and thionine as the monomer. The prepared poly(thionine)-Au composite exhibited an admirable electrochemical redox signal at −0.15 V and excellent H_2_O_2_ catalytic ability. In addition, gold nanoparticles in this composite were found to directly immobilise antibodies and further improve conductivity. In addition, a label-free electrochemical immunosensor was developed using poly(thionine)-Au as the sensing substrate for ultrasensitive detection of cytokeratin antigen 21-1 (CYFRA 21-1), an immunoassay found in human serum. The prepared immunosensor showed a wide liner range from 100 ng mL^−1^ to 10 fg mL^−1^ and an ultralow detection limit of 4.6 fg mL^−1^ (the ratio of signal to noise (S/N) = 3). Additionally, this method was used to analyse human serum samples and yielded results consistency with those of ELISA, implying its potential application in clinical research. The poly(thionine)-Au composite can be easily extended to other polymer-based nanocomposites, which is significant for other electrochemical immunoassays.

## Introduction

Early detection of cancer, one of the leading causes of death worldwide, is critical for successful treatment of the disease and to increase patient survival rates^1–3^. Tumour markers are chemical substances related to cancers, and their determination plays an important role in early detection^4–11^. To date, great efforts have been made to detect such markers, including enzyme-linked immunosorbent assay (ELISA), fluorescent immunoassay, and chemiluminescence enzyme immunoassay^12–16^. Although these methods offer some advantages, there are numerous inevitable drawbacks, such as enzyme deactivation and time-consuming processes. Thus, considerable attention has been devoted to develop a label-free electrochemical immunoassay, due to its desirable properties, including high sensitivity, efficiency, low cost, and user-friendly instrumentation^17–24^.

In a label-free electrochemical immunosensor, the following three aims are extraordinarily important for the sensing substrate: (1) adhering the redox species; (2) enhancing electrochemical signal; and (3) immobilising antibodies. Above all, redox species are indispensable for label-free electrochemical immunosensors and can be implemented in the following three ways^25–33^. One method is by adding the redox species into electrolyte solutions. However, high concentrations of redox species potentially decrease the bioactivity of antibodies or antigens. Redox species can also be modified directly on the substrate by chemical bonds. Yet, this will make the modification process of electrode complicated and tedious. Alternatively, redox species can be adsorbed onto an electrode and covered by a polymer film. The drawback here is the possibility of redox species leakage, which would affect the stability of the label-free electrochemical immunosensor. Although previous studies have introduced enzyme catalysis to achieve a higher electrochemical signal, this could increase the complication and expenses of the immunosensor preparation^34–36^. Considering the above situations, it is of great significance to develop a new type of multifunctional substrate that can provide a redox signal, amplify the signal, and immobilise antibodies for use in a label-free electrochemical immunosensor.

Herein, we synthesised a novel multifunctional poly(thionine)-Au nanocomposite using HAuCl_4_ as the oxidising agent and thionine as the monomer. The as-prepared nanocomposites displayed excellent redox activity and H_2_O_2_ catalytic ability, signal amplification, antibody immobilisation, and good conductivity with a strong single electrochemical redox signal at −0.15 V. Based on these outstanding properties, the poly(thionine)-Au nanocomposite was used as the sensing substrate to develop a label-free electrochemical immunosensor. Cytokeratin antigen 21-1 (CYFRA 21-1) was chosen as the model analyte to be detected. The proposed immunosensor exhibited superior performance, and the detection results were in good agreement with those of ELISA.

## Results and Discussion

**Figure 1:**
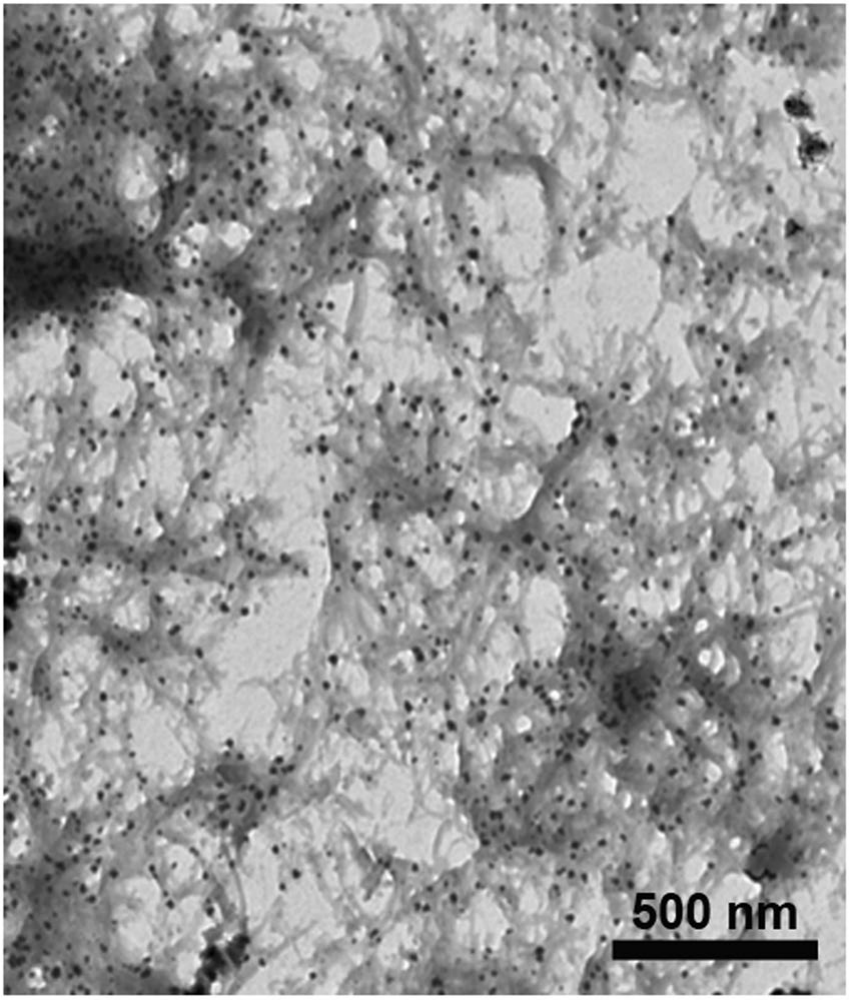
TEM images of poly(thionine)-Au nanocomposite.

The TEM images were used to investigate the morphology of poly(thionine)-Au nanocomposite. In Fig. 1, the gold nanoparticles (AuNPs) are uniformly distributed on poly(thionine). The chemical composition of poly(thionine)-Au was analysed by XPS, and the overview spectrum in Figure S1A reveals the presence of C, N, O, S, and Au atoms in the composite. The carbon component is attributed to the backbone of the benzene ring, and the N is ascribed to secondary amine groups or tertiary amino groups in the composite. Further, the Au 4 f doublet (84.1 and 87.8 eV) in Figure S1B is consistent with the Au° state. These results demonstrate that poly(thionine)-Au was successfully formed.

**Figure 2:**
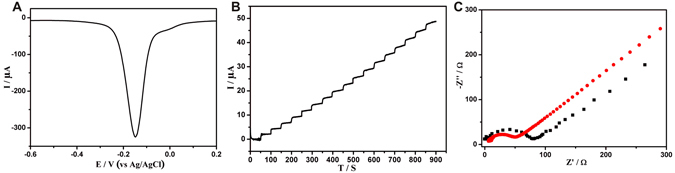
The poly(thionine)-Au nanocomposite exhibited strong redox activity at −0.15 V (**A**), ameperometic response of poly(thionine)-Au for successive addition of 5 mM H_2_O_2_ (**B**), and the EIS of bare GCE (curve a), GCE modified with poly(thionine)-Au (curve b) (**C**).

In addition, the electrochemical redox activity, catalytic ability of poly(thionine)-Au toward H_2_O_2_, and its conductivity were investigated. As shown in Fig. 2A, poly(thionine)-Au exhibited a strong electrochemical signal at −0.15 V, suggesting that it is preconditioned for use as an immunosensing substrate in a label-free electrochemical immunosensor. In addition, amperometric i-t was conducted at −0.1 V in 30 mL PBS in which the current response increased after adding 5 mM H_2_O_2_ into electrolyte solution (Fig. 2B), which indicates that poly(thionine)-Au exhibited excellent H_2_O_2_ catalytic ability. Electrochemical impedance spectroscopy (EIS) was used to investigate the interfacial properties of the electrode modified with the poly(thionine)-Au composite. The Nyquist plot of the poly(thionine)-Au-modified glassy carbon electrode (GCE) (Fig. 2C, curve b) shows a semicircle with a smaller diameter compared to the bare GCE (Fig. 2C, curve a), verifying good conductivity of the nanocomposites.

Poly(thionine) has been used in various electrochemical sensors due to its admirable electrochemical signal and good H_2_O_2_ catalytic ability, and thionine can be polymerised into poly(thionine)-Au using HAuCl_4_ as the oxidizing agent^37, 38^. According to our results, the composite possessed the following outstanding advantages: (1) poly(thionine) displayed strong current signal at −0.15 V and excellent H_2_O_2_ catalytic ability; (2) AuNPs can immobilise the antibody and further improve the electron transfer ability; and (3) the fabrication of this composite only requires an easy one-step method. Based on these merits, poly(thionine)-Au nanocomposite is a promising substrate for a label-free electrochemical immunoassay.

**Figure 3:**
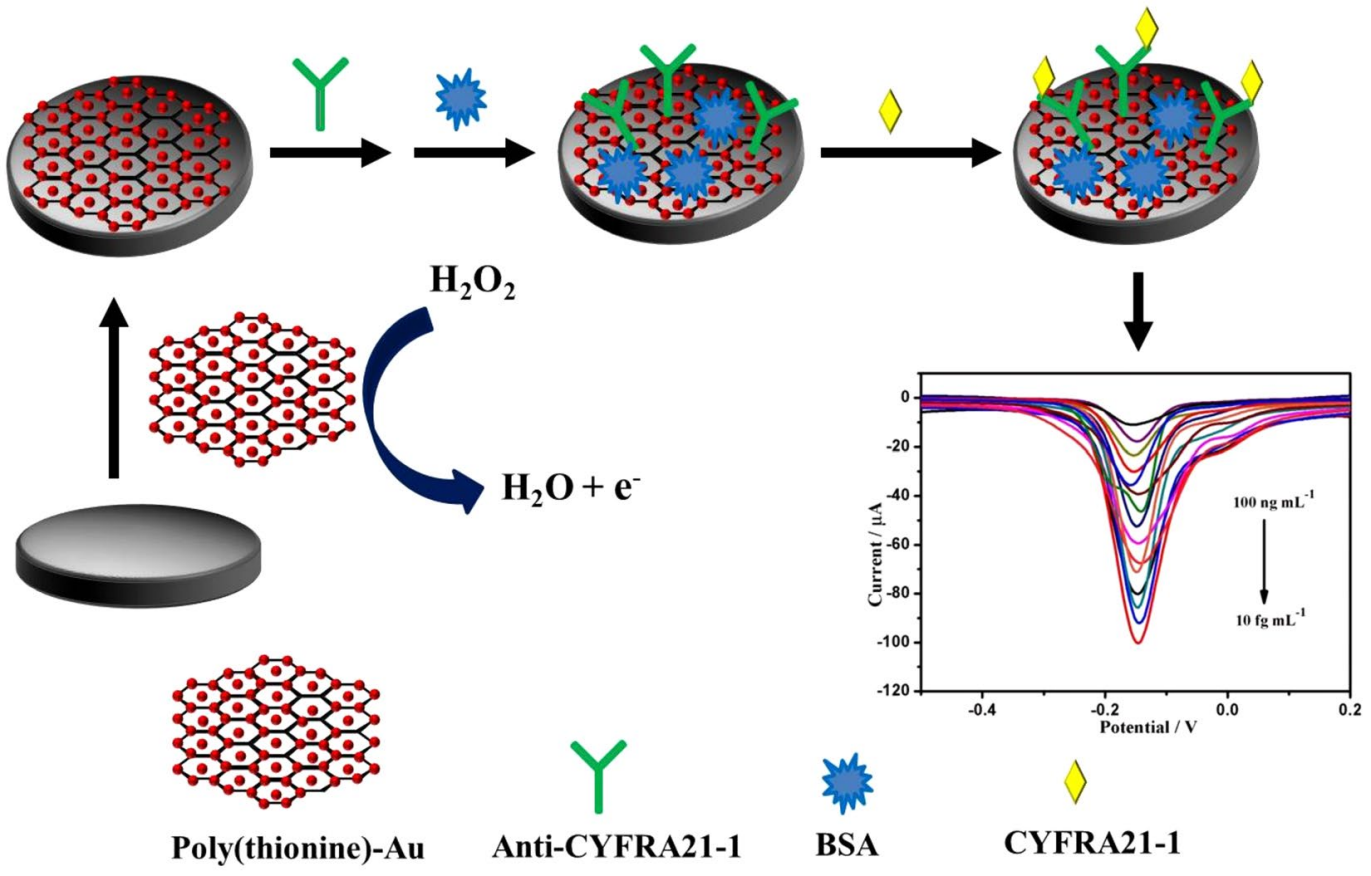
Schematic illustration of the fabrication process of the immunosensing interface.

Herein, the drop-coating method was used to apply the poly(thionine)-Au film onto the glassy carbon electrode (GCE) (Fig. 3). The film was found to catalyse H_2_O_2_ and enhance the current signal at −0.15 V. Following that, the modified electrode was used to adsorb anti-CYFRA 21-1, and excessive antibodies were removed by ultrapure water. After blocking with BSA, a label-free electrochemical immunosensor for CYFRA 21-1 was obtained. The current responses at −0.15 V were proportionate to the logarithm values of CYFRA21-1 concentrations.

**Figure 4:**
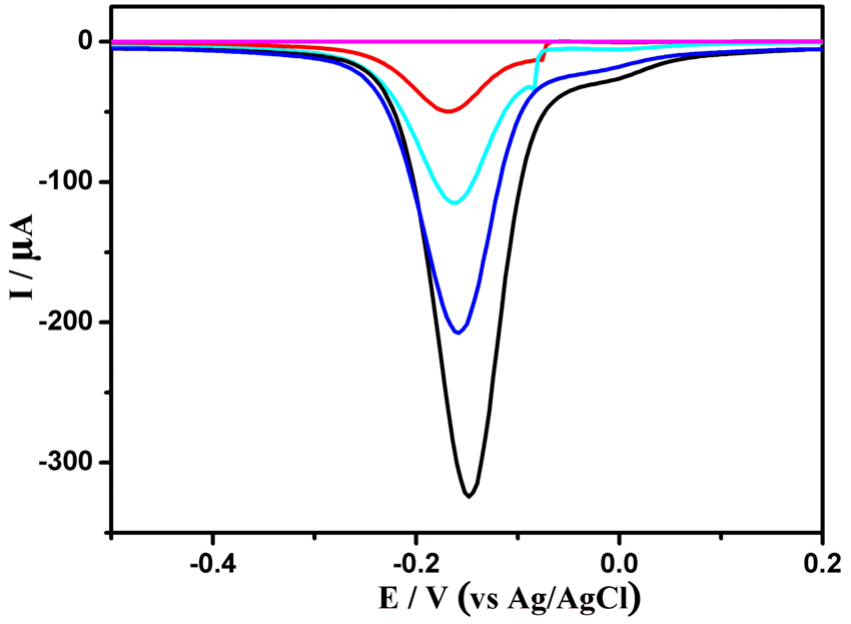
SWV responses of the modified procedure of electrodes in 0.1 M PBS and 5 mM H_2_O_2_ (pH 7.0). (**a**) bare GCE; (**b**) poly(thionine)-Au nanocomposite modified GCE; (**c**) anti-CYFRA21-1/poly(thionine)-Au nanocomposite modified GCE; (**d**) blocked with 1% BSA; (**e**) modified glassy carbon electrode after incubation with 0.5 ng mL^−1^ CYFRA21-1.

Square wave voltammetry (SWV) measurements were used to monitor the electrochemical behaviour of the modification procedure after each step in a phosphate buffer saline (PBS) containing 5 mM H_2_O_2_. In Fig. 4, the bare GCE (curve a) did not display an electrochemical signal, but a strong current response of −0.15 V was obtained after modifying the electrode with poly(thionine)-Au (curve b). Subsequently, the loading of anti-CYFRA 21-1 led to an obvious decrease in the peak current (curve c), owing to the formation of an electron-blocking layer. The current response further decreased after the immunosensor was blocked with BSA (curve d) and incubated in a solution of 0.5 ng mL^−1^ CYFRA 21-1 (curve e). This originated from the insulating layers of BSA and CYFRA 21-1 protein on the electrode, which retarded the electron transfer.

Considering that incubation time directly influences the combination of antibody and antigen, the effect of the incubation time on the immune reaction was investigated. As shown in Figure S2A, the peak current increased with the increase in incubation time, and then kept constant after 50 min. In this case, 50 min was used as the incubation time for the immunoassay. In order to optimise the incubation pH, the effect of pH on the immune reaction was also studied. As shown in Figure S2B, the varied current (∆I) increased when the pH value was decreased to less than 7.0 and decreased when the pH value increased to greater than 7.0. Thus, a pH value of 7.0 was used in subsequent experiments.

**Figure 5:**
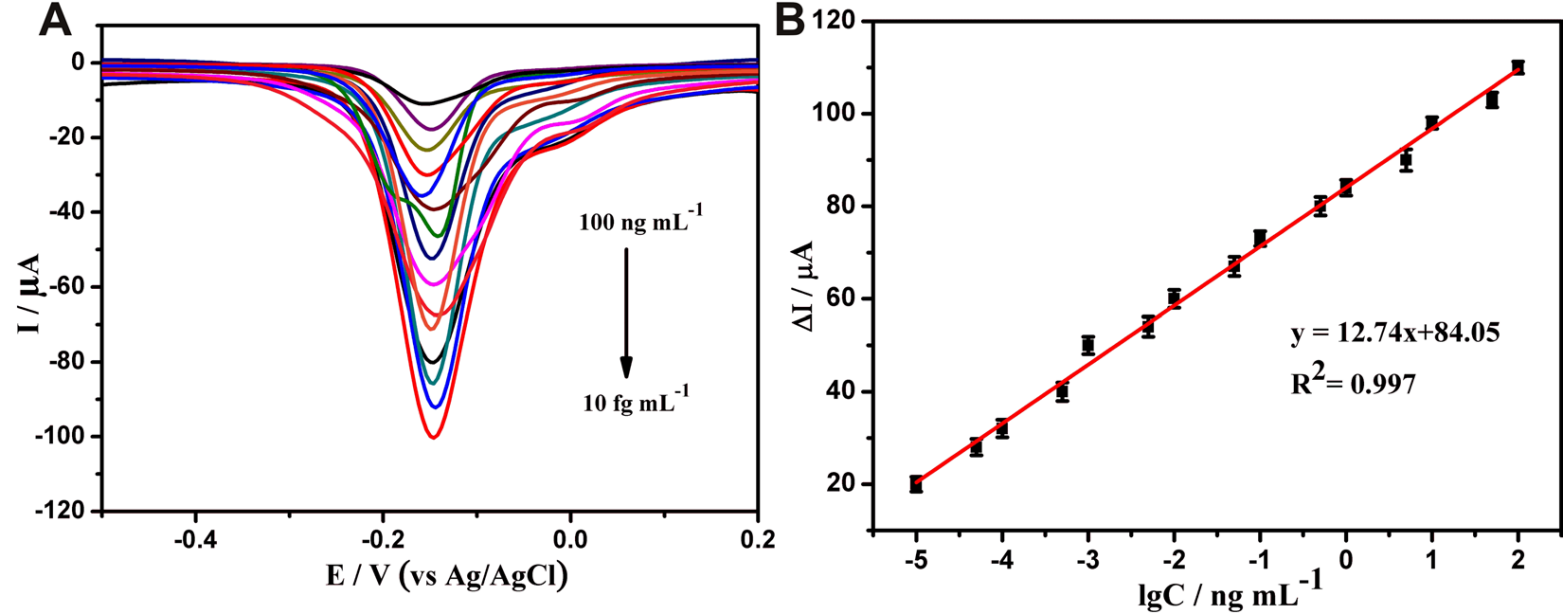
SWV responses of electrochemical immunoassay in 0.1 M PBS and 5 mM H_2_O_2_ (pH 7.0), curves a–i correspond to CYFRA21-1 at the concentrations from 100 ng mL^−1^ to 10 fg mL^−1^. (**B**) The calibration plot between the SWV peak current and the logarithm values of CYFRA21-1 concentrations. Error bars represent standard deviation, n = 3.

The analytical performance of this immunosensor was also tested by SWV. In this experiment, 5 mM H_2_O_2_ was added into the electrolyte solution for signal amplification. The current response of the immunosensor decreased with increasing concentrations of CYFRA 21-1 (Fig. 5A). The immunosensor displayed good linear relation ranging from 100 ng mL^−1^ to 10 fg mL^−1^ with an ultralow detection limit of 4.6 fg mL^−1^ (S/N = 3). Compared with the previous reports (Table S1), it was found that the poly(thionine)-Au-based immunosensor exhibited higher sensitivity, a wider linear range, and lower detection limit. The exceptionally fast response and high sensitivity are attributed to the strong electrochemical signals, excellent H_2_O_2_ catalytic ability, and further improvement in the conductivity due to AuNPs.

To investigate the reliability and accuracy of the above linear relationship, the CYFRA 21-1 content of twenty human serum samples (20 μL each sample) was measured three times with both this immunoassay and ELISA, and the results are shown in Table 1. It can be seen that the relative standard error was less than 8%, indicating good reliability and accuracy of this method.

**Table 1:** Assay results of clinical serum samples using the proposed method and ELISA.

Sample	Proposed immunosensor (ng mL^−1^)	ELISA (ng mL^−1^)	Relative error (%)
1	0.80	0.81	−1.23
2	1.30	1.27	2.36
3	0.60	0.65	−7.69
4	0.60	0.61	−1.64
5	0.60	0.61	−1.64
6	0.75	0.74	1.35
7	0.80	0.82	−2.44
8	0.75	0.74	1.35
9	0.90	0.90	0.00
10	0.90	0.85	5.89
11	0.90	0.91	−1.10
12	0.95	0.90	5.96
13	1.05	1.03	1.94
14	0.95	0.91	4.34
15	1.00	0.99	1.01
16	0.75	0.74	1.35
17	0.75	0.77	−2.50
18	0.65	0.65	0.00
19	0.70	0.74	−5.40
20	0.60	0.61	−1.64

**Figure 6:**
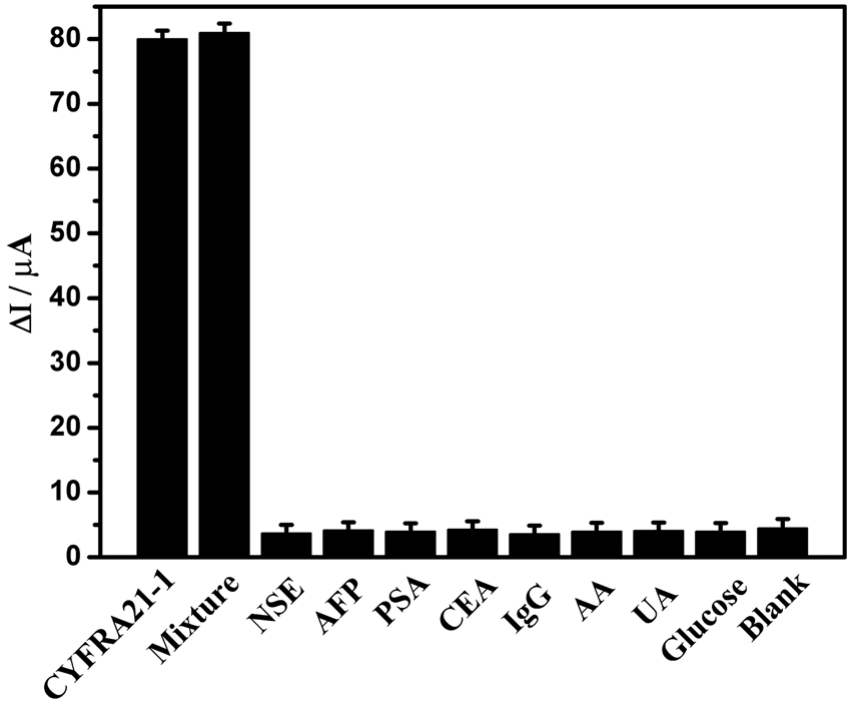
Anti-reference ability of the immunoassay (The error bars are standard deviations for n = 3). The concentrations of IgG, AA, UA, glucose, CEA, PSA, NSE and AFP were 10 ng mL^−1^. The mixture contains IgG (10 ng mL^−1^), AA (10 ng mL^−1^), UA (10 ng mL^−1^), glucose (10 ng mL^−1^), CEA (10 ng mL^−1^), PSA (10 ng mL^−1^), NSE (10 ng mL^−1^) AFP (10 ng mL^−1^), and CYFRA21-1 (0.5 ng mL^−1^).

Selectivity is an important characteristic and it is necessary to test it for the present immunoassay. Numerous analytes, NSE, AFP, BSA, IgG, PSA, AA, and CEA, were used to further test the selectivity of the immunosensor. When the immunosensor was incubated with 10 ng mL^−1^ NSE, AFP, BSA, IgG, PSA, AA, and CEA solution, no obvious changes in the current were observed compared to the blank test (no target analyte) in the same testing conditions, as shown in Fig. 6. However, when CYFRA 21-1 coexisted with interferences, the electrochemical responses were almost the same as that with only CYFRA 21-1. All results indicate that the proposed immunosensor has good specificity for CYFRA 21-1. To test the reproducibility of the immunosensor, five electrodes were prepared for the detection of 0.5 ng mL^−1^ CYFRA 21-1. The relative standard deviation (RSD) of the five electrodes was determined to be 5.3%, suggesting that the reproducibility of the proposed immunosensor is adequate. To further examine the stability of the immunoassay, the immunosensor was stored at 4 °C for about four weeks, and then its electrochemical property was measured. The change in current responses was less than 10%, revealing that the stability of the immunosensor is acceptable (Figure S3).

## Conclusion

In this work, a multifunctional substrate was synthesised using HAuCl_4_ as the oxidising agent and thionine as the monomer. The as-prepared composite exhibited admirable electrochemical redox activity at −0.15 V, excellent H_2_O_2_ catalytic ability, and easy antibody immobilisation. The poly(thionine)-Au nanocomposite was used as sensing substrate to fabricate a label-free electrochemical immunosensor, and the ultrasensitive detection of CYFRA 21-1 was realised. This immunoassay displayed good sensitivity in a wide detection range from 100 ng mL^−1^ to 10 fg mL^−1^ and an ultralow detection limit of 4.6 fg mL^−1^ (S/N = 3). Further, the immunosensor produced consistent results with those of ELISA for detection of human serum samples. The present method is significant for preparing and utilising other label-free amperometric immunosensors using poly(thionine)-Au.

## Methods

### Materials

Ascorbic acid (AA), and hydrogen tetrachloroaurate hydrate (HAuCl_4_·xH_2_O, 99.9%) were purchased from Alfa Aesar China (Tianjin). Mouse anti human monoclonal antibody to cytokeratins antigen 21-1 (anti-CYFRA21-1), CYFRA21-1, neuron-specific enolase (NSE), carcinoembryonic antigen (CEA), alpha fetoprotein (AFP), prostate specific antigen (PSA), were obtained from Shanghai Linc-Bio Science Co., Human immunoglobulin G (IgG), was obtained from Chengwen Biological Company (Beijing, China). Bovine serum albumin (BSA), thionine, hydrochloric acid (HCl, 36.0–38.0%), KCl, NaH_2_PO_4_, Na_2_HPO_4_, K_3_Fe(CN)_6_, K_4_Fe(CN)_6_, were purchased from Beijing Chemical Reagents Company (Beijing, China). Clinical human serum samples were obtained from Capital Normal University Hospital (Beijing, China). All other reagents were of analytical grade and used without further purification. Ultrapure water was used in all experiments (resistivity = 18 MΩ).

### Apparatus

All electrochemical measurements were carried out on a CHI832 electrochemical workstation (Chenhua Instruments Co., Shanghai, China). Transmission electron microscopy (TEM) images were obtained from a JEOL-100CX electron microscope under 80 kV accelerating voltage (H7650, Hitachi, Japan). X-ray photoelectron spectroscopy (XPS) analysis was obtained from an ESCALAB 250 X-ray photoelectron spectroscope (Thermofisher, American). Ultrapure water used in all procedures was purified through an Olst ultrapure K8 apparatus (Olst, Ltd.). A three electrochemical system in the experiment was composed of a glassy carbon electrode (GCE) (4 mm in diameter) as the working electrode, a platinum wire and an Ag/AgCl electrode as counter electrode and reference electrode, respectively.

### Synthesis of poly(thionine)-Au

To synthesise the poly(thionine)-Au nanocomposite, thionine aqueous solution (1%, w/w) and HAuCl_4_ (4%, w/w) were mixed and stirred vigorously for 3 hours at 26 °C. The resulting mixture was then collected by centrifugation at 16000 rpm for 15 min and washed three times with ultrapure water.

### Fabrication of immunosensor

Prior to the functionalisation procedure, a GCE with a diameter of 4 mm was polished using 0.05 μm alumina slurry and followed by ultrasonic cleaning several times in deionized-distilled water. After that, 15 μL dispersion solution of poly(thionine)-Au was dropped onto the surface of the pretreated GCE and allowed to form a thin homogeneous film for 30 min. Next, 80 μL anti-CYFRA21-1 (200 μg mL^−1^) was incubated on the modified electrode at 37 °C overnight. Finally, the resulting modified electrode was further incubated with a solution of BSA (1%, w/w) for 1 h at 37 °C to block the remaining active sites from non-specific absorption. The modified electrode was thoroughly rinsed with ultrapure water, and the desired immunosensor was finally obtained and stored at 4 °C prior to use.

## Electronic supplementary material

Supplementary information
