# Supplementary material for: Multifunctional substrate of label-free electrochemical immunosensor for ultrasensitive detection of cytokeratins antigen 21-1
Source: Sci Rep. 2017 Apr 21;7:1023. doi: 10.1038/s41598-017-01250-0 (PMC5430772; doi:10.1038/s41598-017-01250-0)
Supplement: Supplementary file 1 — Supplementary information [file 41598_2017_1250_MOESM1_ESM.doc]

Supplementary Information

**Multifunctional substrate of label-free electrochemical immunosensor for ultrasensitive detection of cytokeratins antigen 21-1**

Huiqiang Wang, Xin Gao and Zhanfang Ma*

Department of Chemistry, Capital Normal University, Beijing 100048, China


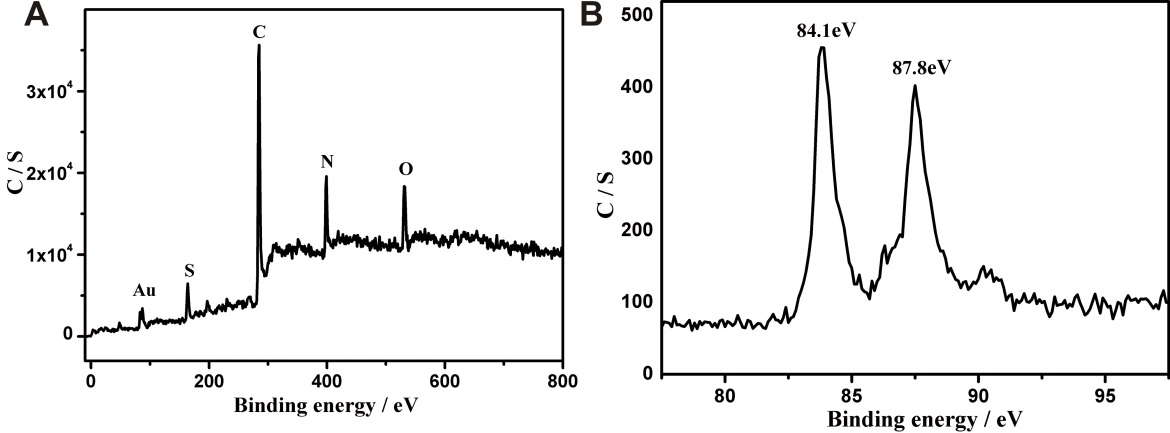


**Figure S1**. XPS spectra of poly(thionine)-Au (A) and High-resolution XPS spectrum of Au4f (B).


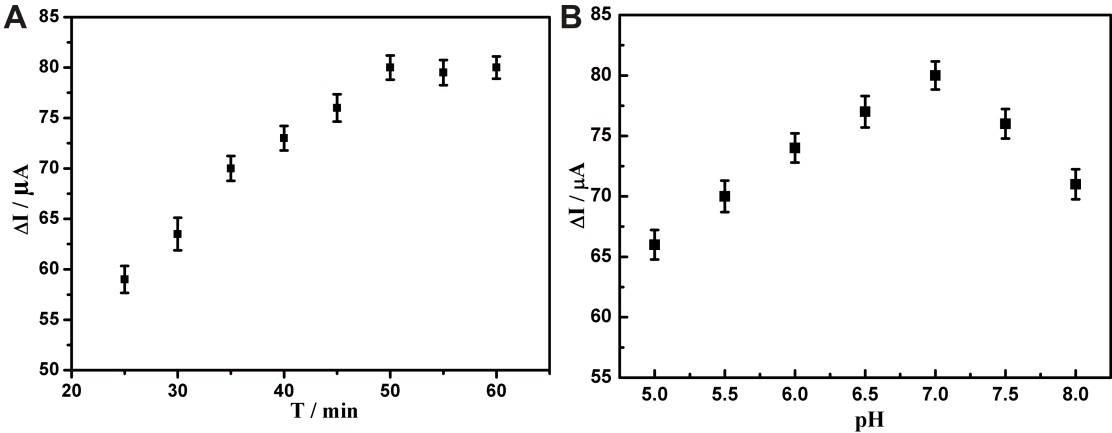


**Figure S2**. Effects of the incubation time (A) and pH (B) on the SWV responses of the immunosensor toward 0.5 ng mL^-1^ CYFRA 21-1.


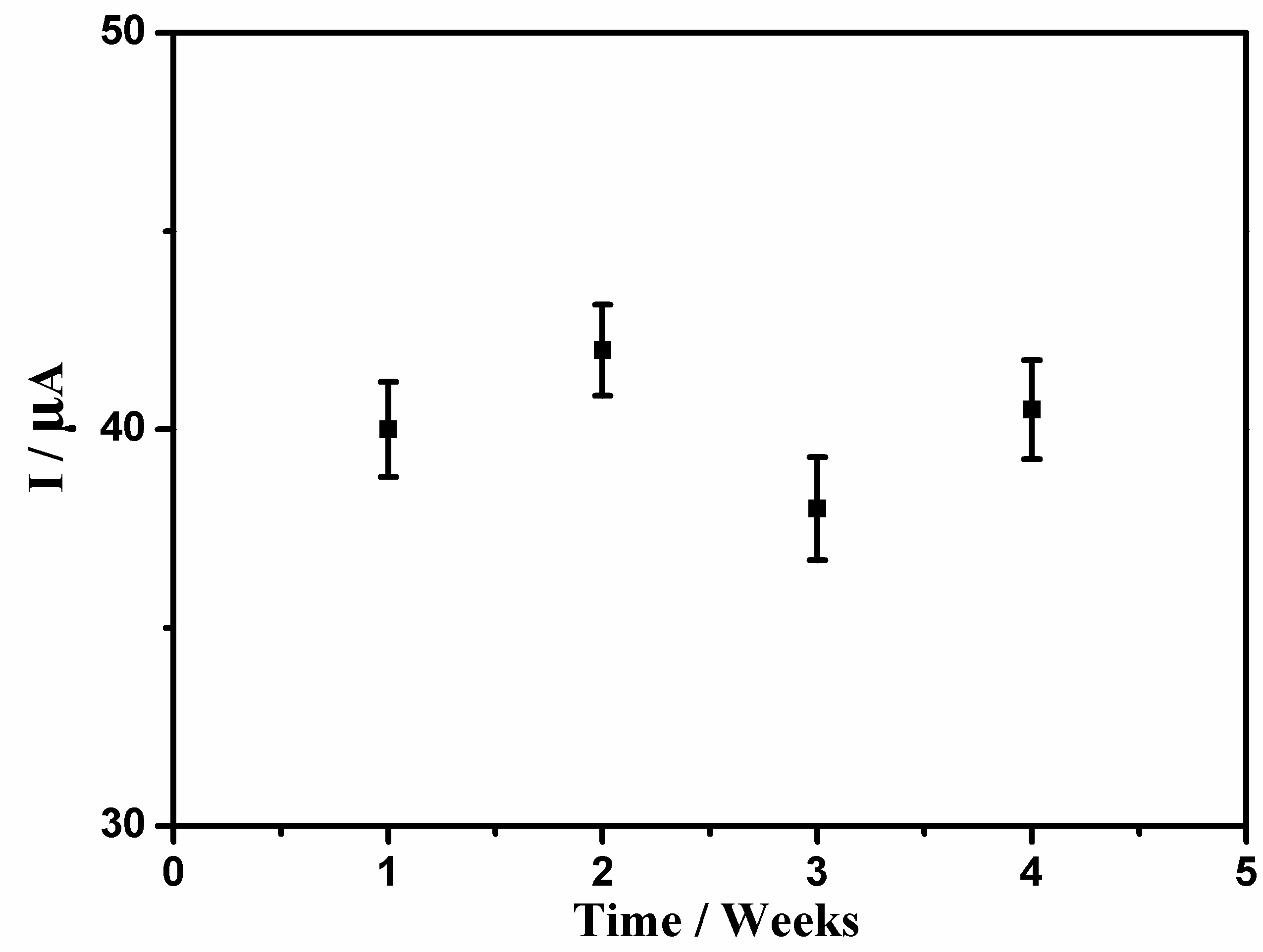


**Fig. S3**. The stability of the immunosensor for the detection of this immunosensor incubated with 0.5 ng mL^-1^ CYFRA 21-1.

**Table S1 A comparison of the performance of the present and referenced immunosensors for the detection of CYFRA 21-1.**

| Substrate materials | Detection method | Linear range  (ng mL^-1^) | Detection limit (pg mL^-1^) | Reference |
| --- | --- | --- | --- | --- |
| Au-PpPD | Electrochemical assay | 0.01-100 | 8.5 | [1] |
| rGO&PHQ-Au | Electrochemical assay | 0.01-200 | 2.3 | [2] |
| PTBO-Au | Electrochemical assay | 1-150 | 400 | [3] |
| Poly(thionine)-Au | Electrochemical assay | 0.00001-100 | 0.0046 | This work |

Annotation: The full name of Au-PpPD is gold-poly(p-phemylenediamine); The full name of rGO&PHQ-Au is polyhydroquinone-graphene composite; The full name of PTBO-Au is poly (toluidine blue o)-gold.

**References**

1. Wang, L. Y., Liu, N., Ma, Z. F., Novel gold-decorated polyaniline derivatives as redox-active species for simultaneous detection of three biomarkers of lung cancer. *J. Mater. Chem. B* **3**, 2867-2872 (2015).

2. Wang, H.Q., Rong, Q. F., Ma, Z. F., Polyhydroquinone-graphene composite as new redox species for sensitive electrochemical detection of cytokeratins antigen 21-1. *Sci. Rep.* **6**, 30623; doi: 10.1038/srep30623 (2016).

3. Shan, J., Ma, Z. F., Simultaneous detection of five biomarkers of lung cancer by electrochemical immunoassay. *Microchim Acta* **183**, 2889-2897 (2016).
